# Depression and anxiety symptoms and their association with quality of life in patients with colorectal cancer: A cross-sectional study

**DOI:** 10.4102/sajpsychiatry.v32i0.2639

**Published:** 2026-06-29

**Authors:** Akmal J.F. Nasution, Elmeida Effendy, Muhammad S. Husada, Mustafa M. Amin, Kamal B. Siregar

**Affiliations:** 1 Department of Psychiatry, Faculty of Medicine, Universitas Sumatera Utara, Medan, Indonesia

**Keywords:** colorectal cancer, depression, anxiety, quality of life, social support

## Abstract

**Background:**

Colorectal cancer is one of the most common types of cancer worldwide, including in Indonesia, and has a significant impact on patients’ quality of life. In addition to the physical burden it causes, colorectal cancer patients often experience psychological disorders such as depression and anxiety, which worsen their quality of life.

**Aim:**

Although many studies have assessed the physical impact of cancer, the relationship between psychological disorders and quality of life still requires further attention.

**Setting:**

This study was conducted at H. Adam Malik General Hospital in Medan, Sumatra.

**Methods:**

This study used a correlative analytical design with a cross-sectional approach, involving 300 colorectal cancer patients. Data were collected using the Patient Health Questionnaire-9 (PHQ-9), Hospital Anxiety and Depression Scale-Anxiety subscale (HADS-A) and World Health Organization Quality of Life-Brief (WHOQOL-BREF) questionnaires to evaluate depression syndrome, anxiety levels and quality of life. Spearman’s test, logistic regression and linear regression model were used in this study.

**Results:**

The results of the study show that 41% of patients experienced severe depression, while 58% of patients experienced severe anxiety. There was a strong negative correlation between symptoms of depression (PHQ-9) and anxiety (HADS-A) and quality of life (WHOQOL-BREF), with a *p*-value < 0.001 for both. Clinical factors such as stage IV and metastasis, as well as socioeconomic status such as employment and education, were also found to have a significant association.

**Conclusion:**

Psychological disorders, particularly depression and anxiety, significantly affect the quality of life of colorectal cancer patients. Psychological interventions, such as cognitive behavioural therapy (CBT), are essential to reduce symptoms of depression and anxiety and improve patients’ quality of life.

**Contribution:**

Strong social support has also been shown to play an important role in improving patients’ psychological well-being and treatment outcomes.

## Introduction

Colorectal cancer is the third most common cancer in men and the second most common in women, and its incidence continues to rise worldwide. On the other hand, thanks to advances in screening, surgical techniques and multimodal therapy, the proportion of patients who survive has increased over the past 20 years. The 5-year survival rate for patients diagnosed with localised disease exceeds 85%.^1^ According to data from the Global Cancer Observatory (GLOBOCAN) 2022, colorectal cancer has the fourth highest incidence worldwide, with more than 1.14 million new cases and 538 167 deaths in 2022.^2^ At the regional level, Asia recorded 531 841 cases of colorectal cancer, accounting for 46.6% of the global total. Asia also contributed 46.2% of the total deaths from colorectal cancer worldwide, with 248 902 deaths in the same year. These figures indicate a significant health burden in Asia related to colorectal cancer, highlighting the need for improved screening and early detection, as well as better management to reduce mortality from this disease.^2^ In Indonesia, colorectal cancer is the second most common type of cancer in men (21 903) and the fourth most common in women (13 773), with a total of 35 676 new cases. Colorectal cancer is also the fifth leading cause of cancer deaths in Indonesia, with 19 255 deaths or 7.9% of total cancer deaths. In addition, there are 104 235 5-year prevalence cases, indicating the long-term burden of this disease on the national health system.^2^ Cancer diagnoses, including colorectal cancer, are often accompanied by a heavy psychological burden. Cancer patients not only face life-threatening physical conditions but also deep emotional stress. Studies show that between 9% and 36.7% of colorectal cancer patients experience symptoms of depression, but only a small proportion receive adequate psychological care.^3^ A meta-analysis conducted by Yuan et al. and colleagues in 2024 examined the relationship between depression and mortality in colorectal cancer patients. The study analysed data from four cohort studies and found that depression was associated with a significantly increased risk of mortality.^4^ The prevalence of depression increases significantly in almost all types of cancer and is the most common mental disorder in cancer patients. The level of depression in cancer patients varies not only based on the type of cancer but is also influenced by various additional factors, including the method of assessing depression, patient demographics, and the course of the cancer. Higher levels of depression are usually found at the end of life, and there is a significant overlap between the burden of physical symptoms and depression. In general, 25% – 30% of patients experience depression in various forms (percentages range from 4% to 60%) during cancer treatment. Additionally, based on a meta-analysis of 70 studies in 14 countries, the combined prevalence of major depression diagnoses in cancer patients is 16.3%.^5^ A 2022 meta-analysis by Cheng concluded that colorectal cancer patients have a 51% increased risk of depression compared with the cancer-free population.^6^ A study conducted by Virginia in 2022 found that 215 colorectal cancer patients awaiting elective surgery showed that 7.9% experienced severe depression, and 21.9% experienced mild to moderate symptoms of depression.^7^ Anxiety in colorectal cancer patients is a significant psychological problem that affects patients’ quality of life. A meta-analysis published in 2024 showed that the prevalence of anxiety symptoms in colorectal cancer patients ranged from 1.0% to 47.2%, with an average of 18.9%. These anxiety symptoms often appear after diagnosis and can persist for a long time, affecting various aspects of patients’ lives.^8^ Factors contributing to high levels of anxiety in colorectal cancer patients include clinical characteristics such as cancer stage, type of treatment (e.g. chemotherapy, radiotherapy or colostomy), and demographic factors such as age and gender.^6^ In addition, psychosocial factors such as social support and psychological resilience also play an important role. Research shows that patients with high levels of psychological resilience tend to have lower levels of anxiety.^9^ The impact of anxiety on colorectal cancer patients is not limited to psychological aspects but can also affect clinical outcomes. Research shows that high anxiety can increase the overall risk of death in colorectal cancer patients.^10^ In addition, anxiety can also reduce adherence to treatment and follow-up care, as well as decrease the overall quality of life of patients. Therefore, it is important for healthcare providers to screen for and intervene early in the symptoms of anxiety in colorectal cancer patients to improve their clinical outcomes and quality of life. This study aims to assess depression syndrome and anxiety symptoms, and their association with quality of life in patients with colorectal cancer, as well as factors that affect depression in colorectal cancer patients.

## Research methods and design

This study is a correlational analytical study with a cross-sectional approach, which evaluated the relationship between depression syndrome, anxiety levels, and quality of life in colorectal cancer patients. The study was conducted at H. Adam Malik General Hospital in Medan, which has been the only national referral centre for cancer treatment on the island of Sumatra since 2014. The sampling technique used in this study was non-probability consecutive sampling. Initially, 335 subjects were approached, but 35 subjects were excluded. A total of 300 subjects were collected according to the inclusion and exclusion criteria. Subjects were recruited based on specific eligibility criteria. The inclusion criteria were: (1) patients with a confirmed diagnosis of colorectal cancer based on medical records; (2) aged between 25 and 60 years; (3) currently receiving treatment at H. Adam Malik General Hospital; (4) capable of communicating and completing questionnaires; and (5) providing written informed consent. The exclusion criteria included: (1) patients with severe cognitive impairment or delirium; (2) a history of pre-existing severe psychiatric disorders; (3) terminal medical conditions that precluded the interview process; and (4) incomplete questionnaire data. Subject recruitment was conducted over a period of 3 months from February to April 2024. In addition to collecting demographic data on the subjects, assessments were conducted using validated Indonesian-language versions of the Patient Health Questionnaire-9 (PHQ-9), Hospital Anxiety and Depression Scale-Anxiety subscale (HADS-A) and World Health Organization Quality of Life-Brief (WHOQOL-BREF) to evaluate depression syndrome, anxiety levels and quality of life in colorectal cancer patients. These instruments have been previously tested and have demonstrated good validity and reliability in the Indonesian population. The PHQ-9 was used to assess depressive symptoms. The PHQ-9 consists of 9 items, each scored from 0 to 3, with a total score ranging from 0 to 27. Depression severity was categorised as mild (5–9), moderate (10–14), moderately severe (15–19) and severe (20–27). Anxiety symptoms were assessed using the HADS-A, which consists of 7 items with a total score ranging from 0 to 21. Anxiety severity was categorised as normal (0–7), borderline abnormal (8–10) and abnormal (11–21). Quality of life was measured using the WHOQOL-BREF, which comprises 26 items across four domains: Physical health, psychological health, social relationships and environment. Each domain score is transformed to a scale of 0–100, with higher scores indicating better quality of life. For descriptive analysis, the overall WHOQOL-BREF transformed score was categorised into five levels of quality of life: very poor (0–20), poor (21–40), moderate (41–60), good (61–80) and very good (81–100). These categories were based on the transformed overall score derived from the four WHOQOL-BREF domains. In the correlational and regression analyses, the continuous overall WHOQOL-BREF transformed score was used. Spearman’s test was used to examine correlations between variables, and logistic regression was used to analyse factors affecting depression syndrome and quality of life in colorectal cancer patients, followed by a linear regression model for the WHOQOL-BREF score. A *p*-value < 0.05 was considered statistically significant.

## Results

This study involved 300 colorectal cancer patients with an average age of 49.26 years (Table 1). The majority of patients were male (58%) and had a high school education (62%). A total of 75 patients used a stoma, with 8% of them using a permanent stoma. The most common cancer stages were stage III (35%) and stage IV (30%), while 35% of patients had metastasis. Severe depression was found in 41% of patients, and severe anxiety was experienced by 58% of patients. The quality of life of patients was mostly rated as poor (43%) or moderate (29%), with only 17% rating it as good.

**TABLE 1:** Demographic characteristics of research subjects.

Variables	Mean ± s.d.	Mean	s.d.	Median (min–max)	*n*	%
Age	49.26 ± 10.079	-	-	-	-	-
**Gender**	-	-	-	-	-	-
Men	-	-	-	-	174	58.0
Women	-	-	-	-	126	42.0
**Education**	-	-	-	-	-	-
Elementary	-	-	-	-	24	8.0
Junior High	-	-	-	-	36	12.0
High School	-	-	-	-	186	62.0
Academy or Higher	-	-	-	-	54	18.0
**Work Status**	-	-	-	-	-	-
Employed	-	-	-	-	72	24.0
Unemployed	-	-	-	-	228	76.0
**Marital status**	-	-	-	-	-	-
Married	-	-	-	-	261	87.0
Unmarried	-	-	-	-	39	13.0
Duration of illness (months)	-	25.28	4–36	-	-	-
**Stoma Use**	-	-	-	-	-	-
Yes	-	-	-	-	225	75.0
No	-	-	-	-	75	25.0
**Stoma type (*n* = 75)**	-	-	-	-	-	-
Permanent	-	-	-	-	24	8.0
Temporary	-	-	-	-	51	17.0
**Cancer Stage**	-	-	-	-	-	-
Stage I	-	-	-	-	30	10.0
Stage II	-	-	-	-	75	25.0
Stage III	-	-	-	-	105	35.0
Stage IV	-	-	-	-	90	30.0
**Metastasis**	-	-	-	-	-	-
Yes	-	-	-	-	105	35.0
No	-	-	-	-	195	65.0
**Chemotherapy**	-	-	-	-	-	-
Yes	-	-	-	-	189	63.0
No	-	-	-	-	111	37.0
**Radiotherapy**	-	-	-	-	-	-
Yes	-	-	-	-	144	48.0
No	-	-	-	-	156	52.0
**Depression Severity (PHQ-9)**	-	-	-	-	-	-
Mild	-	-	-	-	33	11.0
Moderate	-	-	-	-	42	14.0
Moderate–Severe	-	-	-	-	102	34.0
Major	-	-	-	-	123	41.0
**Anxiety Severity (HADS-A)**	-	-	-	-	-	-
Normal	-	-	-	-	15	5.0
Borderline abnormal	-	-	-	-	111	37.0
Abnormal	-	-	-	-	174	58.0
**Quality of Life (WHOQOL BREF)**	-	-	-	-	-	-
Very bad	-	-	-	-	33	11.0
Bad	-	-	-	-	129	43.0
Moderate	-	-	-	-	87	29.0
Good	-	-	-	-	51	17.0
Very good	-	-	-	-	0	0.0

PHQ-9, Patient Health Questionnaire-9; HADS-A, hospital anxiety and depression scale-anxiety subscale; WHOQOL-BREF, World Health Organization Quality of Life-Brief; s.d., standard deviation.

The study found a strong negative correlation between depressive syndrome (measured by PHQ-9) and quality of life (measured by WHO-QOL BREF) in colorectal cancer patients (*r* = −0.819, *p* < 0.001) (Table 2). This means that the degree of depressive syndrome increases as the quality of life decreases. Similarly, a strong negative correlation was observed between the level of anxiety (measured by HADS-A) and quality of life (*r* = −0.745, *p* < 0.001), indicating that higher anxiety levels are associated with lower quality of life in these patients.

**TABLE 2:** Relationship between depressive syndrome (PHQ-9) and anxiety level (HADS-A) with quality of life (WHOQOL-BREF) in colorectal cancer patients.

Variables	*n*	*R*	*p*-value
PHQ-9 with WHOQOL-BREF	300	−0.819	< 0.001
HADS-A with WHOQOL-BREF	300	−0.745	< 0.001

PHQ-9, Patient Health Questionnaire-9; HADS-A, hospital anxiety and depression scale-anxiety subscale; WHOQOL-BREF, World Health Organization Quality of Life-Brief.

This study presents risk factors associated with symptoms of depression and poor quality of life (Table 3). Patients who are unemployed, have a low level of education, have metastatic cancer, or are in stage IV show a higher odds ratio (OR) for depression and reduced quality of life. Moderate-to-severe depression and severe anxiety are significantly correlated with reduced quality of life.

**TABLE 3:** Risk factors for depression and low quality of life.

Predictor	OR (Depression)	95% CI	*p*-value (Depression)	OR (Low QOL, score <60)	95% CI (QOL)	*p*-value (QOL)
Gender (women)	1.34	0.89–2.00	0.154	-	-	-
Age ≥ 50	1.12	0.76–1.64	0.360	-	-	-
Unemployed	1.89	1.20–2.95	0.005	2.01	1.33–3.05	0.002
Low education	1.56	1.03–2.37	0.036	1.79	1.10–2.89	0.019
Permanent stoma	2.10	1.14–3.86	0.016	2.56	1.40–4.66	0.003
Metastatic cancer	2.73	1.70–4.38	0.001	3.25	1.95–5.43	< 0.001
Stage IV	3.11	1.95–5.00	< 0.001	3.60	2.20–5.90	< 0.001
Moderate-to-severe depression	-	-	-	4.81	3.15–7.34	< 0.001
Severe anxiety	-	-	-	4.26	2.90–6.48	< 0.001

OR, odds ratio; QOL, Quality of Life; CI, confidence interval.

This study presents the results of multiple linear regression analysis to predict quality of life (Table 4). Symptoms of depression (PHQ-9) were the strongest predictor (*β* = −0.64), followed by anxiety (HADS-A) and clinical status such as metastasis and stage IV. All predictors in the model were statistically significant (*p* < 0.05).

**TABLE 4:** Multiple linear regression analysis of World Health Organization Quality of Life-Brief scores.

Predictor	*β*	s.e.	Beta (Standardised)	*t*	*p*-value
PHQ-9 (Score > 10)	−1.43	0.21	−0.64	−6.81	< 0.001
HADS-A (Anxiety Score)	−0.97	0.18	−0.41	−5.39	< 0.001
Stage IV (vs Stages I–III)	−4.28	1.12	−0.28	−3.82	< 0.001
Metastasis (Yes)	−5.12	1.30	−0.26	−3.94	< 0.001
Low education (≤ HS)	−2.65	1.08	−0.19	−2.46	0.015
Unemployed	−3.21	1.15	−0.22	−2.79	0.006

PHQ-9, Patient Health Questionnaire-9; HADS-A, hospital anxiety and depression scale-anxiety subscale; WHOQOL-BREF, World Health Organization Quality of Life-Brief; s.e., standard error.

## Discussion

The results of this study confirm previous findings. Demographically, the study population was almost middle-aged (mean age 49.26 years), with a gender distribution of 58% male and 42% female. These results are in line with research by Makmun et al. in Jakarta, who also found similar proportions in terms of age and gender groups.^11^ In a study conducted by Aminisani et al. on the differences in psychological impact between men and women in colorectal cancer patients, it was found that although the prevalence of anxiety was higher in women (57.4%) than in men (50.3%), the rate of depression in men (38.3%) was slightly higher than in women (32.9%). These findings indicate that although psychological disorders such as anxiety and depression affect both genders, women tend to be more prone to anxiety, while men show slightly higher levels of depression. This underscores the importance of a gender-sensitive approach to care, with more routine psychological screening and tailored treatment based on the emotional and psychological differences between men and women.^12^ Our analysis showed that women with colorectal cancer are 1.34 times more prone to have depression and anxiety, although it is not a statistically significant difference. However, we did not conduct subgroup analyses of these variables, underscoring the need for such analyses in further studies. Furthermore, this study found that 41% of patients experienced severe depression, while 58% experienced severe anxiety, indicating the high psychological burden faced by colorectal cancer patients. It was also found that individuals who were unemployed, had a low level of education, or had a clinical status such as metastasis or stage IV cancer had a higher odds ratio (OR) for experiencing depression and a decline in quality of life. Factors such as low education and employment status also influence anxiety and depression levels in patients. Research by Jeleff et al. identified that individuals with low education levels were more likely to have a more fatalistic perception of cancer and were less exposed to information about cancer risk factors, which could influence their decision to undergo cancer screening.^13^ The prevalence of depression and anxiety in our study was quite high. It may be associated with disease stage and treatment status since most of our subjects were in advanced stages of colorectal cancer (stages III and IV) and were receiving chemotherapy without further assessment of their chemotherapy regimen. Further studies may analyse the impact of the chemotherapy regimen on depression and anxiety in colorectal cancer. Various studies show that psychological disorders such as depression and anxiety can worsen the physical and emotional condition of cancer patients, reduce compliance with treatment, and decrease overall quality of life. A study conducted by Yuan et al. shows that depression and anxiety have a long-term impact on the quality of life of colorectal cancer patients and increase the risk of mortality.^4^ We found that depression and anxiety may worsen quality of life in colorectal cancer patients, leading to a hypothesis that it may be correlated with poor social support (social relationships), since it is one of the aspects that were included in WHOQOL-BREF. Lakshmi et al. found that most cancer patients had depression, ranging from mild to severe depression, and they had the lowest score of social relationships in the WHOQOL-BREF score among other aspects, indicating that they had emotional expression and interpersonal communication impairment because most of them had social isolation and withdrawal due to stigma about cancer.^14^ Good social support plays an important role in improving the quality of life of colorectal cancer patients. Patients with strong social support, whether from family, friends or the community, show lower levels of anxiety and depression and better clinical outcomes. The importance of psychosocial support is highlighted in a study by Lian et al., which emphasises the importance of supportive care for elderly colorectal cancer patients undergoing chemotherapy, showing that their care needs continue to increase as chemotherapy cycles progress, while their quality of life and social support experience a significant decline. Although physical support and information are important, psychosocial factors such as emotional support and anxiety management also greatly influence patient well-being.^15^ However, our study did not assess further how social support may improve their quality of life, and it can be considered as a variable that should be assessed in future studies. The results of this study indicate that symptoms of depression (PHQ-9) are the strongest predictor of quality of life in colorectal cancer patients, followed by anxiety (HADS-A) and clinical status such as metastasis and stage IV. All predictors in this model were statistically significant (*p* < 0.05). A meta-analysis by Xia et al. highlights that depression and anxiety are independent factors that affect the survival of colorectal cancer patients. This study found that patients with depression had a higher risk of death compared to those who did not experience depression. Although anxiety is also associated with an increased risk of death, its effect is smaller than that of depression.^10^ Similarly, a study conducted by Alwhaibi et al. used data from the United States: National Health Interview Survey (NHIS) to explore the impact of depression and anxiety on the quality of life of cancer patients. The results showed that patients with depression and anxiety had lower physical and mental quality of life scores than cancer patients without psychological disorders. Patients with both conditions (depression and anxiety) had the lowest quality of life scores.^16^ In addition to patient-related and clinical factors, the healthcare context may also contribute to these findings. The findings of this study should be interpreted within the context of the care provided at H. Adam Malik General Hospital, a national referral centre in Sumatra. The standard of care in this setting primarily emphasises multimodal clinical interventions, including surgery, chemotherapy and radiotherapy. Although these approaches reflect comprehensive physical management, structured psychological screening and consultation-liaison psychiatric services are not yet routinely implemented for all oncology patients.

The high prevalence of severe depression (41%) and anxiety (58%) observed in this study may indicate that patients’ psychological needs are not fully addressed within the current care framework. However, as this study did not directly assess the level or quality of care received, this interpretation should be made with caution. Nevertheless, these findings highlight the potential importance of integrating mental health services into routine oncology care. In this context, ‘comprehensive management’ should extend beyond advanced medical treatment to include systematic psychological assessment and the involvement of mental health professionals as part of a multidisciplinary care approach. Based on existing findings, it is recommended that psychological interventions, such as cognitive behavioural therapy (CBT), become an integral part of colorectal cancer patient management, although we did not test CBT as an intervention in this study. This approach is not only effective in reducing symptoms of depression and anxiety often experienced by patients, but it can also help reduce the side effects of chemotherapy and improve their quality of life. In the study by Wang et al., psychological intervention through CBT therapy was explored as an approach to reduce the side effects caused by chemotherapy and improve immune function in colorectal cancer patients. This therapy was applied to patients receiving chemotherapy to address symptoms of depression and anxiety that are often associated with cancer treatment. Cognitive behavioural therapy can help reduce chemotherapy side effects, such as fatigue and emotional problems, by improving patients’ psychological well-being through relaxation training, stress awareness, self-confidence development and improved coping strategies.^17^ Therefore, further studies may include interventions to assess whether CBT is beneficial for colorectal cancer patients to improve their quality of life through regulating their mental health problems, such as depression and anxiety. This study has several limitations: (1) We did not do an intervention; (2) No multivariate analysis for anxiety; (3) We did not analyse confounding factors of depression and anxiety in colorectal cancer patients.

## Conclusion

Based on the results of this study, a significant relationship was found between symptoms of depression, anxiety and quality of life in colorectal cancer patients. Higher levels of depression and anxiety were associated with poorer quality of life, indicating that psychological distress plays an important role in patients’ overall well-being. In addition, demographic factors such as employment status and education level, as well as clinical conditions including advanced cancer stage and metastasis, were significantly associated with an increased risk of depression and reduced quality of life. Patients with more severe clinical conditions, such as stage IV or metastatic cancer, tended to have a lower quality of life and were more vulnerable to psychological disorders. As a recommendation, it is important to consider the inclusion of psychological interventions in colorectal cancer care, particularly CBT, to help address anxiety and depression. Such approaches may not only reduce psychological symptoms but also improve patients’ quality of life, especially during treatment and recovery. Greater attention should also be given to psychosocial factors, including strong social support, to facilitate recovery and improve clinical outcomes. This study highlights the significant impact of psychological factors on the quality of life of colorectal cancer patients. These findings suggest that integrating psychological interventions and social support into routine oncology care may enhance overall patient outcomes.
